# Experimental and Model-Based Analysis to Optimize Microalgal Biomass Productivity in a Pilot-Scale Tubular Photobioreactor

**DOI:** 10.3389/fbioe.2020.00453

**Published:** 2020-05-13

**Authors:** Tobias Weise, Claudia Grewe, Michael Pfaff

**Affiliations:** ^1^Department of Medical Engineering and Biotechnology, University of Applied Sciences Jena, Jena, Germany; ^2^Department of Bioinformatics, Friedrich Schiller University Jena, Jena, Germany; ^3^BioControl Jena GmbH, Jena, Germany; ^4^Salata AG, Ritschenhausen, Germany

**Keywords:** microalgal cultivation, coarse-grained modeling, light limitation, temperature, biomass productivity optimization, *Nannochloropsis*

## Abstract

A dynamic coarse-grained model of microalgal growth considering light availability and temperature under discontinuous bioprocess operation was parameterized using experimental data from 15 batch cultivations of *Nannochloropsis granulata* in a pilot-scale tubular photobioreactor. The methodology applied consists of a consecutive two-step model parameter estimation using pooled, clustered and reorganized data to obtain initial estimates and multi-experiment fitting to obtain the final estimates, which are: maximum specific growth rate μ_max_ = 1.56 d^−1^, specific photon half-saturation constant *K*_S,ph_ = 1.89 ${\text{mol}}_{\text{ph}}{{\text{g}}_{\text{X}}}^{-1}{\text{d}}^{-1}$, specific photon maintenance coefficient *m*_ph_ = 0.346 ${\text{mol}}_{\text{ph}}{{\text{g}}_{\text{X}}}^{-1}{\text{d}}^{-1}$ and the cardinal temperatures *T*_min_ = 2.3°C, *T*_opt_ = 27.93°C and *T*_max_ = 32.59°C. Biomass productivity prediction proved highly accurate, expressed by the mean absolute percent error MAPE = 7.2%. Model-based numerical optimization of biomass productivity for repeated discontinuous operation with respect to the process parameters cultivation cycle time, inoculation biomass concentration and temperature yielded productivity gains of up to 35%. This optimization points to best performance under continuous operation. The approach successfully applied here to small pilot-scale confirms an earlier one to lab-scale, indicating its transferability to larger scale tubular photobioreactors.

## 1. Introduction

Microalgae as a phylogenetically non-related group of organisms are adapted to a wide variety of inhabited ecosystems (Hoffmann, 2010; Guarnieri and Pienkos, 2014). The mostly photosynthetically active organisms produce a number of substances, such as carotenoids and fatty acids, that are specific to primary producers. A number of them, such as ketocarotenoids (e.g., astaxanthin) or long-chain ω-3 fatty acids (e.g., eicosapentaenoic acid, C20:5, EPA), are of commercial interest to the food and feed industry (Posten, 2012; Minhas et al., 2016).

In comparison to plants, microalgae are in general structurally less complex and show higher area productivities (Derwenskus et al., 2018). Their gross biochemical composition can be influenced without genetic modifications (Gu et al., 2012; Draaisma et al., 2013). These specific characteristics cause growing interest in autotrophic biotechnological production processes based on microalgae. Their products are of particular interest to markets such as aquaculture, nutraceuticals and cosmetics (Khan et al., 2018; Malcata, 2018). This is accompanied by worldwide growing research efforts (Endres et al., 2018; Garrido-Cardenas et al., 2018; Lippi et al., 2018).

The commercial interest in the genus *Nannochloropsis* has risen since they accumulate high lipid contents per cell, high EPA contents and show comparatively high biomass productivities (Gouveia and Oliveira, 2009; Bartley et al., 2014). This eustigmatophyte has a rather small cell size of 2, …, 4 μm; cells are free-floating and easy to ingest by zooplankton and fish larvae, representing a major reason for its extensive use in aquaculture. As other microalgae, *Nannochloropsis* spp. contain variable contents of proteins (24, …, 52%), lipids (16, …, 50%), EPA (3, …, 5%), and carotenoids (0.4, …, 0.6%) (Chua and Schenk, 2017; Hulatt et al., 2017; Neumann et al., 2018). The biochemical composition shifts as a result of cellular adaptation to changing environmental conditions, such as light intensity, temperature, pH value and nutrient availability (Braun et al., 2014; Poliner et al., 2019). The cellular behavior influenced by these abiotic stimuli has been described for *Nannochloropsis* spp.; in particular effects on growth and biochemical composition have been reported (Pal et al., 2011; Wagenen et al., 2012; Wahidin et al., 2013). Here, the applied photobioreactor system sets specific conditions for the culture, e.g., with respect to photon availability (Schediwy et al., 2019).

Tubular photobioreactors are an important type among closed photobioreactor systems used for the phototrophic production of microalgal biomass worldwide (Takache et al., 2009; Grewe and Griehl, 2013; Karemore et al., 2015; Olaizola and Grewe, 2019). Biomass productivity and hence space-time yield (often referred to as volumetric yield) of a photoautotrophic bioprocess are influenced by many abiotic factors, which also change during the course of the day as well as throughout the cultivation cycle (Bernard et al., 2016). Light availability can vary by orders of magnitude along with biomass concentration, layer thickness and external light intensity and is therefore difficult to control, e. g. in outdoor discontinuous bioprocesses. Temperature can also vary in a wide range of 2, …, 30°C (Zittelli et al., 1999; Ras et al., 2013), in particular at larger scale, while pH is easier to control even in industrial applications.

The work presented here employs a dynamic coarse-grained model to describe the light and temperature dependent specific microalgal growth rate within a pilot-scale tubular photobioreactor in order to finally predict biomass growth and optimize biomass productivity. Within the model used, light-limited growth is described depending on the specific light availability rate *q*_ph_. A similar approach has already been successfully applied to optimize steady-state biomass productivity within a lab-scale tubular photobioreactor under turbidostatic operation (Weise et al., 2019). The present work extends this approach to dynamic microalgal growth within discontinuous processes. While under turbidostatic operation *q*_ph_ can be controlled well, under discontinuous operation it can only be kept within reasonable ranges. This is investigated here for a repeated batch process, with the inoculation biomass concentration *c*_X,0_ and the cultivation cycle time *t*_cyc_ as process parameters that can be influenced during operation.

The current work additionally includes temperature dependencies into the model. Therefore, the empirical *Cardinal Temperature Model with Inflexion* (CTMI), first introduced by Lobry et al. (1991), is employed. The CTMI was already successfully applied to describe microbial and microalgal temperature dependencies (Rosso et al., 1993; Bernard and Rémond, 2012; Barbera et al., 2019).

Therefore, the following growth kinetics model for the description of both dependencies, i. e. for light and temperature, was also used here (Equation 1) (Bernard and Rémond, 2012):

(1) $$\begin{array}{l} \mu \left(q_{\text{ph}},T\right)=\mu_{\text{opt}}\left(q_{\text{ph}}\right)\cdot \phi \left(T\right) \end{array}$$

μ_opt_(*q*_ph_) [d^−1^] (Equation 2) represents the optimum specific growth rate for a specific light availability rate *q*_ph_ [${\text{mol}}_{\text{ph}}{{\text{g}}_{\text{X}}}^{-1}{\text{d}}^{-1}$] at the strain-specific optimum temperature *T*_opt_ [°C], whilst ϕ(*T*) [-] (Equations 5 to 7) describes the influence of the temperature *T* [°C] on the optimum specific growth rate.

(2) $$\begin{array}{l} \mu_{\text{opt}}\left(q_{\text{ph}}\right)=\mu_{\text{max}}\frac{q_{\text{ph}}-m_{\text{ph}}}{q_{\text{ph}}-m_{\text{ph}}+K_{\text{S,ph}}}{|}_{T_{\text{opt}}} \end{array}$$

Equation (2) relies on a *Monod*-like function in which μ_max_ [d^−1^] and *K*_S,ph_ [${\text{mol}}_{\text{ph}}{{\text{g}}_{\text{X}}}^{-1}{\text{d}}^{-1}$] are the maximum specific growth rate and the specific half-saturation constant for photon availability, respectively. The specific photon maintenance coefficient *m*_ph_ [${\text{mol}}_{\text{ph}}{{\text{g}}_{\text{X}}}^{-1}{\text{d}}^{-1}$] represents the light energy used for purposes other than biomass synthesis (Pirt, 1986). This kinetics describes the experimentally observed saturation of the growth rate when light availability increases within the investigated range (Darvehei et al., 2018). Regarding *Nannochloropsis* spp., the literature reports maximum specific growth rates μ_max_ in the range of 0.86, …, 1.6 d^−1^ (Sandnes et al., 2005; Spolaore et al., 2006). Specific photon availability rates *q*_ph_ are often found in the range of 0.25, …, 2.2 ${\text{mol}}_{\text{ph}}{{\text{g}}_{\text{X}}}^{-1}{\text{d}}^{-1}$ (under lit conditions; flat panel photobioreactors) (Zijffers et al., 2010; Kandilian et al., 2014; Janssen et al., 2018).

The biomass-specific photon availability rate *q*_ph_ (Equation 4) is calculated from process and geometrical characteristics, in particular from the biomass concentration *c*_X_ [${\text{g}}_{\text{X}}{\text{m}}^{-3}$], the light intensity at the reactor surface *I*_0_ [${\text{mol}}_{\text{ph}}{\text{m}}^{-2}{\text{d}}^{-1}$], the illuminated reactor projection surface *A* [m^2^] and the total reactor liquid volume *V*_*L*_ [m^3^]. The underlying relationships have been successfully applied and demonstrated in several publications (Bernard, 2011; Kliphuis et al., 2011; Blanken et al., 2016). Equation (4) applies only to light-limited growth conditions and was originally developed considering flat-panel photobioreactors (Schediwy et al., 2019). Therefore, the tubular reactor compartment is considered to be a flat-panel equivalent with an average light path length *l*_∅_ (Equation 3). Since *V*_*L*_ has to be equal in both assumptions, the illuminated reactor projection surface is used for *q*_ph_ calculation in Equation (4).

(3) $$\begin{array}{l} l_{\emptyset }\left(r\right)=\frac{\pi r}{2} \end{array}$$

(4) $$\begin{array}{l} q_{\text{ph}}\left(c_{\text{X}}\right)=\frac{1}{V_{\text{L}}\cdot c_{\text{X}}}\overset{c}{\underset{z=1}{\sum }}I_{0,z}\cdot A_{z} \end{array}$$

Within Equation (4) *q*_ph_ [${\text{mol}}_{\text{ph}}{{\text{g}}_{\text{X}}}^{-1}{\text{d}}^{-1}$] is calculated using the light intensity at the reactor surface *I*_0_ [${\text{mol}}_{\text{ph}}{\text{m}}^{-2}{\text{d}}^{-1}$] of an individual reactor compartment multiplied by the illuminated reactor projection surface *A* [m^2^] of the respective compartment (*z* = 1, …, *c*) summarized over all compartments *c*, related to the total reactor liquid volume *V*_*L*_ [m^3^] and the biomass concentration *c*_X_ [${\text{g}}_{\text{X}}{\text{m}}^{-3}$] (Equation 4).

The temperature dependency ϕ(*T*) (within Equation 1) is described using the CTMI (Equations 5-7):

(5) $$\begin{array}{l} \phi \left(T\right)=\{\begin{array}{lll} 0 & \text{for} & T<T_{\text{min}}\\ \hat{\phi }\left(T\right) & \text{for} & T_{\text{min}}<T<T_{\text{max}}\\ 0 & \text{for} & T>T_{\text{max}} \end{array} \end{array}$$

with

(6) $$\begin{array}{l} \hat{\phi }\left(T\right)\\ =\frac{\left(T-T_{\text{max}}\right){\left(T-T_{\text{min}}\right)}^{2}}{\left(T_{\text{opt}}-T_{\text{min}}\right)\left[\left(T_{\text{opt}}-T_{\text{min}}\right)\left(T-T_{\text{opt}}\right)-\left(T_{\text{opt}}-T_{\text{max}}\right)\left(T_{\text{opt}}+T_{\text{min}}-2T\right)\right]} \end{array}$$

and

(7) $$\begin{array}{l} T_{\text{opt}}>\frac{T_{\text{min}}+T_{\text{max}}}{2} \end{array}$$

Outside *T*_min_ and *T*_max_ [°C], which represent the minimum and maximum temperature of the growth range, no growth is assumed (Equation 5). In order to obtain the inflected asymmetric shape of the CTMI, *T*_opt_ values need to be closer to *T*_max_ as to *T*_min_ (Equation 7). Bernard and Rémond (2012) verified both the above model properties (Equations 5, 7), which are necessary for a successful model application in this context. Optimum growth temperatures are stated in the literature within a range of 20, …, 29 °C (Sukenik, 1991; Bartley et al., 2015; Abirami et al., 2017). However, to the best of our knowledge, no optimum growth temperature for *N. granulata* has been published yet.

Model parameter estimation was carried out in two consecutive steps: Initial parameter estimates were generated in a first step from pooled, clustered and reorganized data of the process. These initial estimates were then used in a second step for the final parameter estimation based on the original experimental time series data.

Based on the parameterized model, a numerical optimization regarding the biomass productivity *Pr* was carried out with respect to the process parameters inoculation biomass concentration *c*_X,0_, cultivation cycle time *t*_cyc_ and temperature *T*. Also, suggestions are provided with respect to the transfer of the proposed approach to other tubular photobioreactors under repeated discontinuous operation.

## 2. Materials and Methods

### 2.1. Cultivation Conditions

The strain of *Nannochloropsis granulata* (Karlson et al., 1996) used in this study was previously isolated and its identity confirmed by 18s rDNA analysis carried out by SAG Göttingen. All experiments were performed using sterile brackish water medium with a salinity of 19 psu, a nitrate concentration of 12 mM and a phosphate concentration of 0.3 mM provided by Prof. Otto Pulz, IGV GmbH, Nuthetal, Germany. The composition of the artificial brackish water medium 1/2 ES1 (*Enriched Seawater*): NaCl 243.0 mM, MgSO_4_ · 7 H_2_O 29.3 mM, NaNO_3_ 16.5 mM, MgCl_2_ · 6 H_2_O 12.7 mM, CaCl_2_ · 2 H_2_O 5.4 mM, KCl 5.3 mM, K_2_HPO_4_ · 3 H_2_O 0.8 mM, FeSO_4_ · 7 H_2_O 43.18 μM, MnCl_2_ · 4 H_2_O 2.02 μM, ZnSO_4_ · 7 H_2_O 0.30 μM, CuCl_2_ · 2 H_2_O 0.23 μM, H_3_BO_4_ 0.16 μM, NaMoO_4_ · 2 H_2_O 0.09 μM and CoCl_2_·6 H_2_O 0.08 μM.

Both, nitrate and phosphate concentrations were measured using ready-to-use cuvette test kits (nitrate: WTW 252073; phosphate: WTW 252075, Xylem Analytics Germany, Weilheim, Germany) after centrifugation of samples at 4, 500*g for 20 min. Cell-free supernatant was diluted with double distilled water 1:100 for nitrate and 1:10 for phosphate analysis according to the manufacturer's instructions. Absorbance values and ion concentrations were determined in a photoLab® 6100 VIS Photometer (Xylem Analytics Germany, Weilheim, Germany). Nitrate and phosphate were added manually on demand from sterile stock solutions of NaNO_3_ (300 gl^−1^) and KH_2_PO_4_ (100 gl^−1^) if values fell below 50% of the initial medium concentrations (${\text{c}}_{{\text{NO}}_{3}^{-}}$ = 1,021 mgl^−1^, ${\text{c}}_{{\text{PO}}_{4}^{-3}}$ = 98 mgl^−1^).

Maintenance cultures of *N. granulata* were kept in cell culture flasks at 14 °C and 50 $\mu {\text{mol}}_{\text{ph}}{\text{m}}^{-2}{\text{s}}^{-1}$ light intensity (12 h light/12 h dark).

Preculturing was carried out in 1.7 l double jacked glass bubble columns (height to diameter ratio of 4), temperated at 22 °C and aerated at 0.1 vvm with a 2% CO_2_ to air ratio (v/v). Precultures where illuminated using fluorescent tubes (Philips Cool White, 36 W, eight tubes per column, horizontally arranged). The light intensities at the inner surfaces of the columns were on average 249 $\mu {\text{mol}}_{\text{ph}}{\text{m}}^{-2}{\text{s}}^{-1}$, measured by a spherical micro quantum sensor (US-SQS, Walz GmbH & Co KG, Ulm, Germany). During preculture, illumination was provided to the columns continuously for 24 h.

The 30 l photobioreactor was inoculated using precultures from four individual bubble columns which were originally inoculated by the same maintenance culture.

### 2.2. Experimental Set-Up

**Figure 1:**
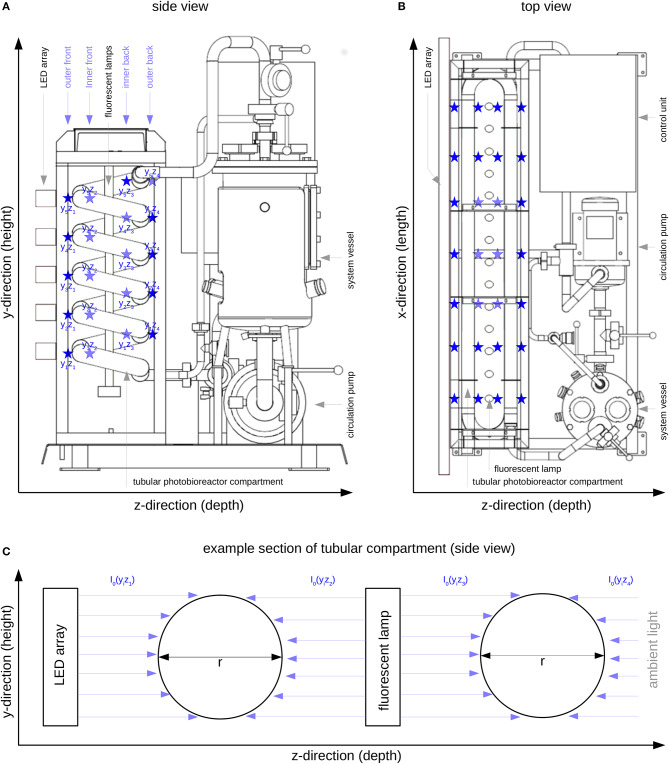
Tubular photobioreactor system PBR30 [IGV Institut für Getreideverarbeitung GmbH, Nuthetal, Germany]; Position of the LED array, the fluorescent lamps and the illuminated tubular reactor part; ⋆ *I*_0_ measuring positions (see also Table 1); **(A)**: Side view; **(B)**: Top view; **(C)**: Representation of four modeled compartments in y/z-direction (referring to Figure 1A), *I*_0_(y_i_z_j_) - surface light intensity of the respective compartment, y_i_ - ith position in y-direction, z_j_ - jth position in z-direction.

The experimental work carried out within this study was conducted using a 30 l pilot-scale tubular photobioreactor (PBR30, IGV GmbH, Nuthetal, Germany) consisting of a compartmented glass tube (borosilicate glass, inner diameter of 39.6 mm) and a stainless steel system vessel (see Figure 1). Illumination was provided permanently over 24 h to the glass tube of the reactor by fluorescent tubes (OSRAM Cool White, L 18 W/840) and five LED panels placed along the length of the compartmented glass tube (Figure 1). The LED panels had a spectral composition consisting of 95% red light (λ = 660 nm) and 5% blue light (λ = 440 nm).

The illumination profile of the reactor's tubular glass part was recorded at 140 measuring positions (indicated by ⋆ in Figure 1 and Table 1) at 7 positions in x-direction (width), 5 positions in y-direction (height) and 4 positions in z-direction (depth). All measurements were carried out using the US-SQS described above. Since only minor variations in the light intensities in x-direction were observed (Figure 1B), values in this direction were averaged (see Table 1). The resulting 20 *I*_0_ values refer to their position in y/z-direction as indicated in Figure 1A. These values were used as surface light intensities of the respective reactor compartments within the modeling approach.

**Table 1:** Upper part: Light intensity at the reactor surface of the compartments used within the modeling approach, ⋆ Measuring positions (compare Figure 1A); Center part: Reactor-specific and strain-specific constants; Lower part: Set parameter constraints.

**Light intensity at the reactor compartments surface**					
***I***_**0**_ [$\mu {\text{mol}}_{\text{ph}}{\text{m}}^{-2}{\text{s}}^{-1}$]					
		z-direction (depth)			
	⋆	1	2	3	4
	5	606	230	210	28
	4	762	323	286	26
y-direction (height)	3	735	392	342	29
	2	836	360	298	21
	1	731	320	185	19
**Reactor-specific and strain-specific constants**					
**Constant**		**Value**		**Unit**	
*r*		0.020		m	
*A*		1.269		m^2^	
*V*_*L*_		0.030		m^3^	
*c*		20		-	
**Set parameter constraints**					
**Parameter**		**Constraint**		**Unit**	
	**Lower**		**Upper**		
μ_max_	1.0		3.8^a^	d^−1^	
*K*_S,ph_	0		1.9	${\text{mol}}_{\text{ph}}{{\text{g}}_{\text{X}}}^{-1}{\text{d}}^{-1}$	
*m*_ph_	0		0.32^b^	${\text{mol}}_{\text{ph}}{{\text{g}}_{\text{X}}}^{-1}{\text{d}}^{-1}$	
*T*_min_	−5		19^*^	°C	
*T*_opt_	−5^**^		50^**^	°C	
*T*_max_	32^*^		50	°C	

* *For explanation see Section 2.6.2 Search Range*.

** *Provided the model's properties are fulfilled (Equations 5, 7)*.

a Sorokin and Krauss (1958);

b *Zijffers et al. (2010)*.

The pH value was controlled by the injection of pure CO_2_ at the intake side of the circulation pump using a limit controller (set-point pH 7.25; manipulated variable: solenoid-controlled valve; switch-point: pH 7.25; pH hysteresis: 0.05; on-delay: 5 s). The temperature was controlled using a cooling water circuit supplied by a water bath (F12, Julabo GmbH, Seelbach, Germany) and connected to the double jacket of the system vessel (limit comparator; hysteresis: 1 K). The circulation pump frequency was set to 35.7 Hz resulting in a suspension flow velocity of 0.72 m s^−1^ in the glass tubes.

### 2.3. Experimental and Modeling Approach

**Figure 2:**
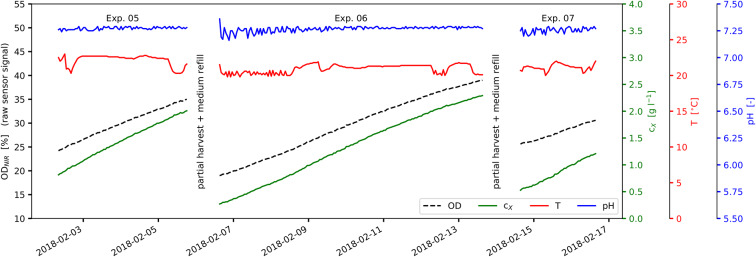
View of experimental process data: Experiments No. 05-07 as part of a series of repeated discontinuous cultivations; experiments are separated by partial harvest and medium refill; *OD*_NIR_, raw sensor signal *OD*_NIR_ (*black*); *c*_X_, correlated biomass concentration (*green*); *T*, suspension temperature (red); *pH*, suspension pH value (*blue*).

The experimental data used within this work was collected from altogether 15 cultivations which were carried out as a series of discontinuous (batch) bioprocesses in order to avert the lag phase at the start of the individual experiments (see Figure 2). Cultures were harvested partially by draining parts of the suspension and replacing it by fresh, steam sterilized medium (Figure 2). The experiments were designed to be conducted at constant illumination at the reactor surface *I*_0_ but by varying the inoculation biomass concentrations *c*_X,0_, the cultivation cycle times *t*_cyc_ and the cultivation temperatures *T* (see Table 2-lower part and **Figures 5A,B**). Temperature variations during the cultivations, however, resulted from a limited cooling capacity of the reactor system under strongly differing ambient conditions.

**Table 2:** Experimental and modeling results: Upper part: Initial Ω_1_, Ω_2_, and final estimates Ω_f_ (mean values and jackknife 95 % confidence intervals (CI), if applicable); Lower part: Experimental values and model predictions, *c*_X_/*OD*_NIR_ correlation parameters.

**Estimates**	**Ω_1_**	**Ω_2_**		**Ω_f_**								
				**Lower CI**		**Mean**		**Upper CI**		**σ**		**Unit**
μ_max_	2.33	1.76		1.54		**1.56**		1.59		0.17		d^−1^
*K*_S,ph_	3.00	2.27		1.84		**1.89**		1.93		0.31		${\text{mol}}_{\text{ph}}{{\text{g}}_{\text{X}}}^{-1}{\text{d}}^{-1}$
*m*_ph_	0.32	0.29		0.344		**0.346**		0.347		0.010		${\text{mol}}_{\text{ph}}{{\text{g}}_{\text{X}}}^{-1}{\text{d}}^{-1}$
*T*_min_	12.7	0.0		1.9		**2.3**		2.8		2.9		°C
*T*_opt_	27.1	28.4		27.88		**27.93**		27.99		0.37		°C
*T*_max_	34.0	32.4		32.53		**32.59**		32.66		0.42		°C
**Data set**	**Experimental values**			**Model prediction results and errors**						***c*_X_*/OD*_NIR_ correlation parameters**		
***j***	***t***_**cyc, j**_	***c***_**X,0,*j***_	***Pr***_*data*, ***j***_	***Pr***_**model**, ***j***_			**(*M*)*APE_*j*_***			***a***	***b***	**R**^**2**^
				**Ω*_1_***	**Ω*_2_***	**Ω*_f_***	**Ω*_1_***	**Ω*_2_***	**Ω*_f_***			
	**[d]**	**[**${\text{g}}_{X}l^{-1}$**]**	**[**${\text{g}}_{X}l^{-1}d^{-1}$**]**	**[**${\text{g}}_{X}l^{-1}d^{-1}$**]**			**[%]**			**[**${\text{g}}_{X}l^{-1}{AU}^{-1}$**]**	**[**${\text{g}}_{X}l^{-1}$**]**	**[-]**
01	3.4	0.89	0.36	0.31	0.32	0.32	12.7	9.7	10.3	0.100	-1.241	1
02	3.4	0.74	0.36	0.31	0.33	0.32	12.4	8.5	9.1	0.092	-1.067	1
03	3.0	0.78	0.38	0.39	0.37	0.37	0.6	3.7	3.9	0.114	-1.816	1
04	2.9	1.03	0.34	0.34	0.34	0.34	1.8	1.8	1.7	0.108	-1.719	1
05	3.5	0.81	0.35	0.36	0.35	0.35	2.1	0.2	0.2	0.111	-1.864	1
06	7.0	0.27	0.29	0.28	0.29	0.29	2.0	1.9	0.5	0.101	-1.650	0.99
07	2.0	0.53	0.34	0.33	0.34	0.33	3.9	1.9	3.3	0.136	-2.965	0.97
08	3.7	0.81	0.34	0.29	0.31	0.31	14.5	7.4	8.4	0.101	-1.058	1
09	0.7	0.98	0.25	0.29	0.32	0.31	12.2	26.1	23.6	0.225	-3.992	1
10	3.4	0.63	0.38	0.37	0.34	0.33	3.9	9.6	13.3	0.094	-1.071	1
11	2.7	0.96	0.28	0.30	0.32	0.32	8.9	17.4	16.1	0.095	-0.985	1
12	7.0	0.46	0.31	0.31	0.31	0.30	0.9	0.7	1.7	0.094	-0.796	0.99
13	7.0	0.48	0.29	0.33	0.32	0.32	14.2	9.7	8.7	0.090	-0.723	0.99
14	7.0	0.43	0.32	0.37	0.34	0.34	15.0	7.7	5.6	0.103	-1.278	0.98
15	6.2	0.66	0.34	0.37	0.34	0.34	7.2	1.0	1.8	0.103	-1.310	0.98
∅			**0.33**	0.33	0.33	**0.33**	7.5	7.2	**7.2**			

In order to carry out the modeling, the photobioreactor was subdivided into 20 glass tube compartments of equal size (*z* = 1, …, 20). Light intensity at the respective compartment surface was mapped as shown in Table 1 and remained constant during each individual experiment and across the experiments.

As described by Weise et al. (2019), *I*_0_ is considered to be a sum parameter for the light field description at the respective compartment surface. According to Equation (4) only light absorption is taken into account. Due to the actual reactor features (see Figure 1C), the light field is considered to illuminate the compartment y_i_z_j_ from only one side with equal intensity. In addition, because of the particular reactor arrangement, as shown in Figure 1C, each physical compartment is represented by two modeled compartments. The resulting photon fluxes (i.e., light intensity multiplied by surface area) within the modeled compartments were added and then related to the total biomass concentration and the reactor volume (see Equation 4).

### 2.4. Data Collection

During the experiments a number of variables were determined on-line and off-line. The on-line values of the optical density at near-infrared *OD*_NIR_, the pH and the temperature *T* were continuously measured and recorded by a 6-channel writer (JUMO Logoscreen 500 cf, Fulda, Germany). The sampling of the off-line values was carried out daily in the morning. The biomass concentration *c*_X_ was determined as salt-free dry cell weight. Samples for the dry cell weight determination were homogenized, an aliquot of 10 ml was taken, centrifuged in pre-weighted glass tubes at 4.500 * g for 20 min; the resulting supernatant was discarded and the biomass pellet resuspended in 9 ml deionized water; this washing step was carried out twice. The pellets were dried at 105 °C for 24 h and weighed after cooling in a desiccator for 45 min. Measurements were carried out in duplicate.

The original experimental time series data are available as Supplementary Material.

### 2.5. Numerical Calculations

All computations were performed using the programming language python (version 3.6.5) and the additional packages astropy (version 3.0.4), numpy (version 1.15.0), pandas (version 0.23.4), SALib (version 1.3.8), scipy (version 1.1.0), and seaborn (version 0.9.0).

#### 2.5.1. Biomass Concentration

*c*_X_ [${\text{g}}_{\text{X}}{\text{l}}^{-1}$] was calculated from on-line *OD*_NIR_ [AU] values using a linear correlation. Parameters *a* [${\text{g}}_{\text{X}}{\text{l}}^{-1}{\text{AU}}^{-1}$] and *b* [${\text{g}}_{\text{X}}{\text{l}}^{-1}$] (see Table 2-lower part) have been estimated from off-line *c*_X_ measurements for each individual experiments (original data not shown).

#### 2.5.2. Averaged Approximated Growth Rate

The calculation of the averaged approximated growth rate ${\bar{\mu }}_{\Delta }$ [d^−1^] is carried out using the approximation of the ordinary differential equation of the biomass concentration for discontinuous bioprocesses (Equation 8). At first, the approximated growth rate μ_Δ_(t) [d^−1^] is calculated employing the central difference approximation (Equation 9), which is subsequently smoothed using a moving average (Equation 10), where *n* is the total number of data points *i* between *t* − 1 h and *t* + 1 h. The employed central difference approximation linearizes the growth between the two observations and is therefore only applicable for small Δ*t* (here: Δ*t* = 2 h).

(8) $$\begin{array}{l} \frac{\Delta c_{\text{X}}}{\Delta t}=\mu_{\Delta }\cdot c_{\text{X}} \end{array}$$

(9) $$\begin{array}{l} \mu_{\Delta }\left(t\right)=\frac{c_{\text{X}}\left(t+1\text{h}\right)-c_{\text{X}}\left(t-1\text{h}\right)}{2\text{h}\cdot c_{\text{X}}\left(t\right)} \end{array}$$

(10) $$\begin{array}{l} {\bar{\mu }}_{\Delta }\left(t\right)=\frac{1}{n}\overset{n=i_{t+1\text{h}}}{\underset{i_{t-1\text{h}}}{\sum }}\mu_{\Delta }\left(i\right) \end{array}$$

#### 2.5.3. Biomass Productivity

The experimental biomass productivity for the respective experiment *j* was calculated as volumetric yield using the following Equation (11) (data point *i*, *i* = 0, n).

(11) $$\begin{array}{l} Pr_{j}=\frac{c_{\text{X},i=\text{n},j}-c_{\text{X},i=0,j}}{t_{i=\text{n},j}-t_{i=0,j}} \end{array}$$

#### 2.5.4. Model Validation by MAPE

The criterion used to evaluate the model's accuracy was the Mean Absolute Percent Error (MAPE) (Equation 12) (Mayer and Butler, 1993). It is calculated based on measured and modeled productivities considering all experiments (*j* = 1, …, *m*). When only a single experiment is considered (*m* = 1), MAPE is expressed as APE (Absolute Percent Error).

(12) $$\begin{array}{l} \text{MAPE}=\frac{100}{\text{m}}\overset{m}{\underset{j=1}{\sum }}\left(\frac{\left|Pr_{\text{meas},j}-Pr_{\text{model},j}\right|}{\left|Pr_{\text{meas},j}\right|}\right) \end{array}$$

### 2.6. Parameter Estimation and Model Analysis

In order to parameterize the developed model, a parameter estimation procedure was carried out in two consecutive steps: The *initial parameter estimation*, following the methodology proposed by Bernard and Rémond (2012), was applied in a first step to generate initial parameter estimates which were then, in a second step, used as starting values for the *final parameter estimation*. Both stages of the consecutive parameter estimation were carried out using the same experimental (time series) data.

The *initial parameter estimation* (section 2.6.3) allows to efficiently screen through a wide parameter search range (see section 2.6.2) by fitting the growth kinetics directly to the reorganized data sets (see section 2.6.1). This was carried out in particular with respect to the temperature modeling in order for the unknown cardinal temperatures to converge. Since, however, the reorganization includes approximation and averaging of ${\bar{\mu }}_{\Delta }$ as well as clustering and selection, the returned results are potentially imprecise.

Therefore, the *final parameter estimation* (section 2.6.4) was carried out by fitting the model output to the original experimental time series data sets, employing the above mentioned initial estimates as starting values.

Based on the model, parameterized this way, the numerical *biomass productivity optimization* (section 2.6.6) was carried out with respect to the process parameters *c*_X,0_, *t*_cyc_, and *T* that are to be applied to the process.

#### 2.6.1. Data Set Reorganization

In order to carry out the *initial parameter estimation* procedure described in the following section, a pooling, reorganization, clustering, and selection of the data was performed:

- Calculating the averaged approximated growth rates ${\bar{\mu }}_{\Delta ,i,j}$ (according to Equation 10) for the *i*^th^ data point of the *j*^th^ experiment (*j* = 1, …, 15).
- Concatenating data sets for *q*_ph,*i,j*_, ${\bar{\mu }}_{\Delta ,i,j}$ and *T*_*i,j*_ from all 15 experiments into a new single data set.
- Reassigning the newly formed entries for ${\bar{\mu }}_{\Delta ,i}$ and *T*_*i*_ that share the same *q*_ph_ value to a new *k*^th^ data set which corresponds to this particular *q*_ph_ value (*k* = 1, …, *s*).
- Sorting each data set *k* obtained by its *T*_*i,k*_ values (for identical *T*_*i,k*_ values, corresponding ${\bar{\mu }}_{\Delta ,i,k}$ values were averaged).
- Clustering the entire *q*_ph_ range into 10 equidistant clusters; assigning each data set *k* to its respective cluster.
- Selecting one data set per *q*_ph_ cluster that covers the widest individual *T*_*k*_ range (*k* = 1, …, 10).

The data sets (*k* = 1, …, 10) reorganized this way were then applied in the first part of the parameter estimation procedure.

#### 2.6.2. Search Range

An adequate search range for the parameter estimation procedure was determined based on reasonable assumptions. In particular, the temperature range was constrained to -5.0, …, 50.0° C, based on the assumption that growth outside this range is not possible for the genus *Nannochloropsis*.

- *T*_min_ ranges from −5.0°C (lower bound) to the lowest temperature for which experimental data is available (upper bound).
- *T*_opt_ ranges from −5.0°C (lower bound) to 50.0 °C (upper bound), as long as the model's properties are fulfilled (see Equations 5, 7).
- *T*_max_ ranges from the highest temperature for which experimental data is available (lower bound) to 50.0 °C (upper bound).
- μ_max_ ranges from the highest ${\bar{\mu }}_{\Delta }$ value obtained from the experiments (lower bound) to the highest μ_max_ value reported for microalgae (upper bound; see Table 1-lower part).
- *K*_S,ph_ ranges from 0 (lower bound) to the highest *q*_ph_ value obtained from the experiments (upper bound).
- *m*_ph_ ranges from 0 (lower bound) to the highest *m*_ph_ value reported in the literature (upper bound; see Table 1-lower part).

#### 2.6.3. Initial Parameter Estimation

Each data set *k*, reorganized as described above, contains growth rate values ${\bar{\mu }}_{\Delta ,i,k}$ at temperatures *T*_*i,k*_. Bernard and Rémond (2012) published a strategy that consists in the following: Identifying *s* + 3 parameters μ_opt,1_, μ_opt,2_, …, μ_opt,*s*_, *T*_min_, *T*_opt_, *T*_max_ (here *s* = 10, for the 10 cluster data sets) vectorised as θ_*T*_, where the parameter μ_opt,*k*_ is the optimum growth rate at the optimum temperature *T*_opt_ for the *k*th data set. Following their strategy, the underlying key assumption is that the cardinal temperatures (*T*_min_, *T*_opt_, *T*_max_) are common to all the data sets, whilst μ_opt,*k*_ is constant within the respective data set due to constant experimental lighting conditions *q*_ph,*k*_. The unknown *s* + 3 parameter values were estimated in a first step by minimizing the optimization criterion *J*(θ_*T*_) (Equation 13) (Bernard and Rémond, 2012). Within a second step, the remaining model parameters μ_max_, *K*_S,ph_ and *m*_ph_ (vectorised as θ_*q*_ph__) were estimated in a similar way (Equation 14). Both, Equations (13) and (14), were minimized with respect to the respective θ resulting in the minimum optimization criterion value *J*(Ω) for the optimum parameter vector Ω according to Equation (15).

(13) $$\begin{array}{l} J\left(\theta_{T}\right)=\overset{s}{\underset{k=1}{\sum }}\overset{n}{\underset{i=1}{\sum }}{\left({\bar{\mu }}_{\Delta ,i,k}-\mu_{\text{opt},k}\cdot \phi \left(T_{i,k},T_{\text{min}},T_{\text{opt}},T_{\text{max}}\right)\right)}^{2} \end{array}$$

(14) $$\begin{array}{l} J\left(\theta_{q_{\text{ph}}}\right)=\overset{s}{\underset{k=1}{\sum }}{\left(\mu_{\text{opt},k}-\mu_{\text{opt}}\left(q_{\text{ph},k},\mu_{\text{max}},K_{\text{S,ph}},m_{\text{ph}}\right){|}_{T_{\text{opt}}}\right)}^{2} \end{array}$$

(15) $$\begin{array}{l} J\left(\Omega \right)=\underset{\theta }{\text{min}}J\left(\theta \right) \end{array}$$

The downhill-simplex method (*Nelder-Mead* method) used for the model parameter estimation procedure (scipy.optimize.fmin) was started from every possible parameter value combination within the search ranges described above. The *T* range was screened stepwise by Δ*T* = 5 °C, resulting in 70 initializations that match the model's properties (Equations 5, 7). The ranges for μ_max_, *K*_S,ph_ and *m*_ph_ were screened using 10 equidistant values along each axis, resulting in 1,000 initializations. The method was set to perform a maximum of 200 iterations for each initialization. Computations were carried out using the standard settings of the above package regarding the convergence criteria of the method.

#### 2.6.4. Final Parameter Estimation

Since the *initial parameter estimation* described above can provide results which are potentially imprecise due to the approximation and averaging of ${\bar{\mu }}_{\Delta }$, the reorganization and clustering of the data sets *k* as well as the split parameter estimation of θ_*T*_ and θ_*q*_ph__, a second and final parameter estimation step was carried out. Based on the ordinary differential equation of the biomass concentration for discontinuous bioprocesses (Equation 16), the original experimental time series data sets *j* (*j* = 1, …, 15) were simulated using the initial parameter estimates. Numerical integration was carried out employing the *Euler* method. The time series of *c*_X_ were simulated for the *i* discrete time points *t*_*i,j*_ and their corresponding temperatures *T*_*i,j*_.

(16) $$\begin{array}{l} \frac{dc_{\text{X}}}{dt}=\mu \left(q_{\text{ph}},T\right)\cdot c_{\text{X}} \end{array}$$

The objective of this *final parameter estimation* was to identify the *m* + 6 parameters *c*_X,0,1_, *c*_X,0,2_,…, *c*_X,0,m_, μ_max_, *K*_S,ph_, *m*_ph_, *T*_min_, *T*_opt_, *T*_max_ (here *m* = 15, for the 15 experiments) vectorised as θ_f_, where the parameter *c*_X,0,*j*_ is the inoculation biomass concentration *c*_X,0_ for the *j*th data set. The optimum parameter values were estimated by minimizing the optimization criterion *J*(θ_f_) (Equation 17). The final optimum parameter vector Ω_f_ (according to Equation 18) was determined in analogy to the initial parameter estimation as described above.

(17) $$\begin{array}{l} J\left(\theta_{\text{f}}\right)=\overset{m}{\underset{j=1}{\sum }}\overset{n}{\underset{i=1}{\sum }}{\left(c_{\text{X,meas}}-c_{\text{X,pred}}\right)}^{2} \end{array}$$

with

(18) $$\begin{array}{l} c_{\text{X},\text{meas}}=c_{\text{X},\text{data},i,j}\\ c_{\text{X},\text{pred}}=c_{\text{X},\text{model},i,j}(q_{\text{ph},i,j},T_{i,j},c_{\text{X},0,j},\mu_{\text{max}},K_{\text{S},\text{ph}},m_{\text{ph}},T_{\text{min}},\\ \;T_{opt},T_{\max})\\ J\left(\Omega_{\text{f}}\right)=\underset{\theta }{\text{min}}J\left(\theta_{\text{f}}\right) \end{array}$$

The *Nelder-Mead* method was also employed for this second step of the model parameter estimation procedure (scipy.optimize.fmin). The initially estimated parameter vectors were now used as start parameter vectors. The method was set to carry out a maximum of 200 iterations for each initialization. Again, the standard settings of the above package were used with respect to the convergence criteria.

Since the values of the parameters *T*_min_ and *T*_max_ to be estimated were expected to lie outside the experimental data range and to account for the relatively small number of experiments, the *Delete-2-Jackknife* analysis (astropy.stats.jackknife_stats) was used to indicate the 95 % confidence interval of the identified parameters of the vector Ω_f_ (Efron and Tibshirani, 1993; Duchesne and MacGregor, 2001). Jackknife re-sampling was carried out by bootstrapping two respective data sets (experiments) from the data at a time.

#### 2.6.5. Parameter Sensitivity Analysis

A variance-based sensitivity analysis according to Sobol (2001) was performed using the SALib.analyze.sobol in order to investigate the model's parameter sensitivities more deeply. This sensitivity analysis was done with respect to the biomass productivity as the main focus of this study. Within Equation (19), *M* represents the model output depending on the process parameters *c*_X,0_, *t*_cyc_ and *T* as well as the model parameter vector θ (Zhang et al., 2017) consisting of μ_max_, *K*_S,ph_, *m*_ph_, *T*_min_, *T*_opt_, and *T*_max_.

The *Sobol* method is based on the decomposition of the variance of the model output *Var*(*M*) (Equation 20) into summands of increasing dimensionality (Zhang et al., 2015, 2017). *Var*_*i*_ is the partial variance corresponding to the first-order index of θ_*i*_ of the model output *M*, while *Var*_*ij*_ is the partial variance corresponding to the second-order index of the ith and jth parameter interaction (Zhang et al., 2015, 2017). *k* is the number of model parameters, here *k* = 6.

(19) $$\begin{array}{l} M=Pr_{\text{model}}\left(c_{\text{X},0},t_{\text{cyc}},T,\theta \right) \end{array}$$

(20) $$\begin{array}{l} Var\left(M\right)=\overset{k}{\underset{i=1}{\sum }}Var_{i}+\overset{k-1}{\underset{i=1}{\sum }}\overset{k}{\underset{j=i+1}{\sum }}Var_{ij}+\ldots +Var_{1,\ldots ,k} \end{array}$$

The sensitivity indices *S*_*i*_ and *S*_*ij*_ (Equations 21, 22) are calculated as ratios of the partial variances to the total variance (Zhang et al., 2015). Total-order indices *S*_*Ti*_ are calculated following Equation (23) using *Var*_~*i*_ which represents the variation of all parameters except θ_*i*_ (Homma and Saltelli, 1996; Sobol, 2001; Zhang et al., 2017). *S*_*i*_ quantifies the effect of varying θ_*i*_ alone, while *S*_*Ti*_ quantifies the effect of varying θ_*i*_ and includes all effects caused by its interactions with all other model parameters.

(21) $$\begin{array}{l} \text{First-order index }S_{i}=\frac{Var_{i}}{Var} \end{array}$$

(22) $$\begin{array}{l} \text{Second-order index }S_{ij}=\frac{Var_{ij}}{Var} \end{array}$$

(23) $$\begin{array}{l} \text{Total-order index }S_{Ti}=S_{i}+\underset{j\neq i}{\sum }S_{ij}+\ldots =1-\frac{Var_{\sim i}}{Var} \end{array}$$

The *Saltelli* sampling scheme (SALib.sample.saltelli) was used to generate model parameter samples of the final model parameter estimates Ω_f_ (section 2.6.4) varied by ±3σ (see Table 2-upper part). This scheme generates *N* · (2*k* + 2) samples (here *N* = 10,000; *k* = 5). The calculated indices were classified using thresholds. Indices contributing <0.01 to the model output variance are considered “non-sensitive,” while indices contributing ≥ 0.1 are considered “highly sensitive” (Tang et al., 2007).

*S*_*i*_ and *S*_*Ti*_ also potentially depend on the process parameters. The experimentally set *c*_X,0_ is associated with the present light availability, while the set *T* may influence the sensitivity of the three cardinal temperatures. Therefore, the sensitivity analysis was carried out for 9 combinations (scenarios) of *c*_X,0_ and *T* taking into account data corresponding to percentages of the respective experimental range [20% (“low”), 50% (“medium”), and 80% (“high”)].

#### 2.6.6. Biomass Productivity Optimization

In order to optimize the biomass productivity *Pr* with respect to the described photobioreactor, a numerical optimization was carried out. The objective was to maximize *Pr* with respect to the process parameters *c*_X,0_, *t*_cyc_, and *T*. Numerical integration of Equation (16) was performed using the estimated model parameters (Ω_f_; Table 2-upper part) by employing lsoda of the ODEPACK (scipy.integrate.odeint). After each integration, *Pr* was calculated for the individual iteration steps using Equation (11). The process parameters *c*_X,0_, *t*_cyc_, and *T* were vectorised as θ_Pr_ containing all possible process parameter combinations, while Ω_Pr_ contains the optimum process parameter combination with respect to *Pr*. The optimum process parameter values were estimated by minimizing the optimization criterion *J*(θ_Pr_) (Equations 24, 25) using also the downhill-simplex method (Nelder-Mead method; scipy.optimize.fmin). With respect to numerical integration and convergence criteria, standard settings of the above packages were used.

(24) $$\begin{array}{l} J\left(\theta_{\text{Pr}}\right)=-Pr_{\text{model}}\left(c_{\text{X},0},t_{\text{cyc}},T\right) \end{array}$$

(25) $$\begin{array}{l} J\left(\Omega_{\text{Pr}}\right)=\underset{\theta }{\text{min}}J\left(\theta_{\text{Pr}}\right) \end{array}$$

## 3. Results and Discussion

The experiments carried out and presented here consisted of a series of discontinuous (batch) cultivations. Single experiments were separated by partial harvest of the biomass suspension and replacement by new medium (see Figure 2). Inoculation biomass concentrations *c*_X,0_ and cultivation cycle times *t*_cyc_ were varied between the experiments (see Figure 2 and Table 2-lower part). Temperature *T* as well as pH were recorded during the experiments (see Figures 2, **5A,B**). The original experimental time series data and the reorganized data sets are available as Supplementary Material.

Light saturation was not observed during the experiments. The linear accumulation of biomass indicates limited lighting conditions (Figure 2). The light intensity at the photobioreactor surface varied between 19 and 836 $\mu {\text{mol}}_{\text{ph}}{\text{m}}^{-2}{\text{s}}^{-1}$ (average: 337 $\mu {\text{mol}}_{\text{ph}}{\text{m}}^{-2}{\text{s}}^{-1}$; see Table 1-upper part and Figure 1). These incident light intensities range from low values (100 $\mu {\text{mol}}_{\text{ph}}{\text{m}}^{-2}{\text{s}}^{-1}$ Raso et al., 2011) to rather typical values at lab-scale (700 $\mu {\text{mol}}_{\text{ph}}{\text{m}}^{-2}{\text{s}}^{-1}$ Pal et al., 2011; up to 850 $\mu {\text{mol}}_{\text{ph}}{\text{m}}^{-2}{\text{s}}^{-1}$ Wagenen et al., 2012) for *Nannochloropsis* sp. With respect to the scalability toward outdoor production, higher incident light intensities of 1,500 to 2,000 $\mu {\text{mol}}_{\text{ph}}{\text{m}}^{-2}{\text{s}}^{-1}$ are present on sunny days. These however are lowered due to the vertical arrangement of the glass tubes at industrial scale, the light absorption by greenhouse parts, etc. Therefore, the comparability and the transferability of presented model to industrial-scale outdoor conditions is limited unless further model extensions regarding higher variability in light and temperature, nightly biomass losses, etc. are carried out.

The inoculation biomass concentrations within this study (0.27,…, 1.03 ${\text{g}}_{\text{X}}{\text{l}}^{-1}$) are within a typical range for both, lab-scale and outdoor production of *Nannochloropsis* sp. in tubular photobioreactors (Olofsson et al., 2012; Pfaffinger et al., 2016; Pereira et al., 2018). The experimental variability regarding *c*_X,0_ is rather high within the investigated small pilot-scale experiments. However, biomass concentrations throughout the cultivation cycle time are found to be typical for large-scale and outdoor production of *Nannochloropsis*. *c*_X_ ranged 0.27,…, 2.79 ${\text{g}}_{\text{X}}{\text{l}}^{-1}$, reflecting usual inoculation and harvesting biomass concentrations, respectively. To the best of our knowledge, depending on the climatic zone, biomass concentrations of *Nannochloropsis* outdoor cultures within tubular photobioreactors rarely exceed 3 ${\text{g}}_{\text{X}}{\text{l}}^{-1}$ (Olofsson et al., 2012; Benvenuti et al., 2015) and do not exceed 5 ${\text{g}}_{\text{X}}{\text{l}}^{-1}$ (Zittelli et al., 1999). Therefore, the *c*_X_ range observed here is comparable to larger-scale and outdoor processes.

Biomass productivity *Pr* was calculated for each experiment according to Equation (11) in order to evaluate the model's accuracy expressed as MAPE (see Table 2-lower part) and to use it for the biomass productivity optimization. The overall context of this work was to optimize *Pr* as part of a series of batch bioprocesses (i.e., repeated batch).

The devised two-stage consecutive parameter estimation procedure was carried out using the reactor-specific and strain-specific constants as well as the set parameter constraints shown in Table 1.

The *initial parameter estimation* converged to two valid solutions (Ω_1_ and Ω_2_, Table 2-upper part) depending on the start parameter combinations. Both solutions differ only strongly in the estimated value of *T*_min_.

Both initial parameter estimation vectors (Ω_1_ and Ω_2_) were used as start parameter combinations for the *final parameter estimation*. It was performed using the original time series data sets *j* (in contrast to the reorganized data sets *k*). The altogether six shared model parameters where estimated within multi-experiment fittings.

**Figure 3:**
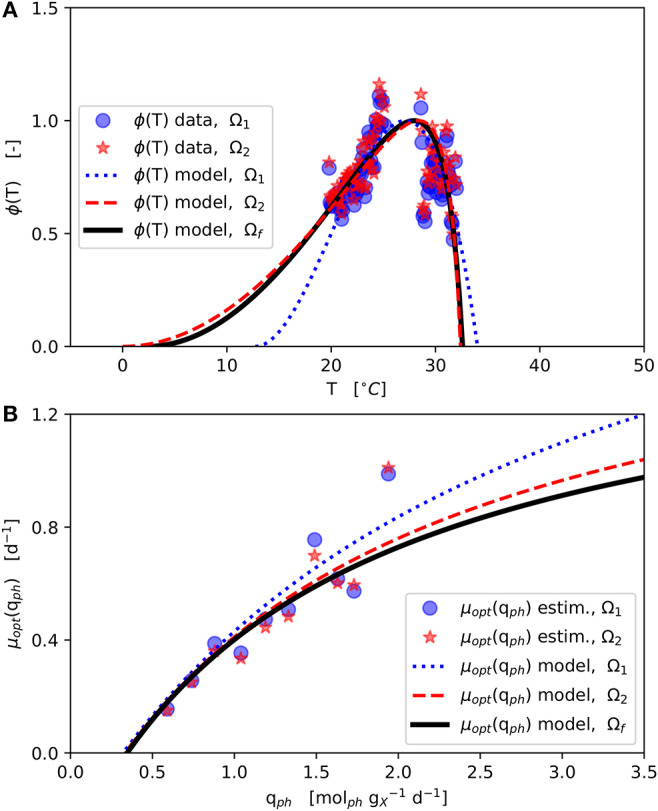
Model parameter estimation results: Initial parameter estimation provides solutions Ω_1_ and Ω_2_ and final parameter estimation solution Ω_f_ (see Table 2–upper part); **(A)**: CTMI estimation ϕ(*T*) vs. *T* (Equations 5–7); **(B)**: Estimation μ_opt_(*q*_ph_) vs. *q*_ph_ (Equation 2).

The *final parameter estimation* converged to the solution Ω_f_ (Table 2-upper part, Figure 3) initialized from each of the two initial parameter combinations (Ω_1_ and Ω_2_). The ϕ(*T*) data shown in Figure 3A was normalized with respect to the corresponding estimated μ_opt_ (Figure 3B) according to Equation (1).

The estimated values of *T*_min_ and *T*_max_ lie outside the range 19, …, 31 °C of available experimental data. Since the range between the estimated *T*_min_ and the available data is much wider than the one between the estimated *T*_max_ and the available data, in particular the *T*_min_ estimation is afflicted with some uncertainty, which is further enforced by the asymmetric shape of the temperature function ϕ(*T*) as can be seen in Figure 3A showing the CTMI results. This is underlined by the jackknife 95 % confidence intervals CI and standard deviations σ of the estimated parameters (Table 2-upper part). While for all other parameters the σ values indicate relatively certain parameter estimations, σ for *T*_min_ is at the same order of magnitude as the parameter's value.

The finally estimated cardinal temperatures for *N. granulata* (*T*_min_ = 2.3°C, *T*_opt_ = 27.93°C, *T*_max_ = 32.59°C) lie within the specified parameter constraint ranges (Table 1) and match laboratory experience. The CTMI parameters estimated by Bernard and Rémond (2012) for the related species *N. oceanica* (data by Sandnes et al., 2005) showing a good degree of similarity (*T*_min_ = −0.2°C, *T*_opt_ = 26.7 °C, *T*_max_ = 33.3 °C).

The results obtained here are supported by an optimum growth temperature range of 20, …, 29 °C given in the literature (Sukenik, 1991; Wagenen et al., 2012; Bartley et al., 2015; Abirami et al., 2017). Further, Wagenen et al. (2012) reported only very poor growth below 13.6 °C and above 32.3°C for *N. salina*. Also, Sukenik (1991) found that *Nannochloropsis* spp. failed to grow below 10 °C and above 38 °C. In view of the fact that the parameter estimation carried out here had only rough quantitative specifications with regard to the cardinal temperatures, the values calculated (Table 2-upper part) are in strong compliance with the literature. Sandnes et al. (2005) empirically described a light-dependent optimum cardinal temperature *T*_opt_ for *N. oceania* (Sandnes et al., 2005). Although this combined effect of light and temperature is physiologically plausible, the functional relationship of these two variables was not observed for *N. granulata* within the own data.

The μ_opt_ values for Ω_1_ and Ω_2_ estimated increase monotonically with higher *q*_ph_ (Figure 3B). These estimates are subject to fluctuations but show no inhibition within the observed *q*_ph_ range. Therefore, a growth kinetics considering light limitation (Equation 2) without light inhibition was applied. The *q*_ph_ range observed during the experiments (0.59, …, 1.9 ${\text{mol}}_{\text{ph}}{{\text{g}}_{\text{X}}}^{-1}{\text{d}}^{-1}$) is comparable to *q*_ph_ values given by the literature (0.25, …, 2.2 ${\text{mol}}_{\text{ph}}{{\text{g}}_{\text{X}}}^{-1}{\text{d}}^{-1}$) (Zijffers et al., 2010; Kandilian et al., 2014; Janssen et al., 2018). In accordance with Equation (4) *q*_ph_ is calculated as a mean light availability rate of the whole culture suspension, considering the total culture volume *V*_*L*_. However, local light intensities differ due to light gradients within the culture suspension, which are not spatially resolved within the model. Despite this aggregation, the devised model predicts microalgal growth and biomass productivities precisely. The estimated photon maintenance coefficient *m*_ph_ = 0.346 ${\text{mol}}_{\text{ph}}{{\text{g}}_{\text{X}}}^{-1}{\text{d}}^{-1}$ is comparable to values reported for *C. sorokiniana*: 0.16 ${\text{mol}}_{\text{ph}}{{\text{g}}_{\text{X}}}^{-1}{\text{d}}^{-1}$ and *D. tertiolecta*: 0.32 ${\text{mol}}_{\text{ph}}{{\text{g}}_{\text{X}}}^{-1}{\text{d}}^{-1}$ (Zijffers et al., 2010). Pirt (1986) reported increasing *m*_ph_ values for photobioreactor set-ups with lower illuminated/non-illuminated culture volume ratios, which has to be considered during the transfer to different photobioreactor set-ups. The growth yield *Y*_X,ph_ [${\text{g}}_{\text{X}}{{\text{mol}}_{\text{ph}}}^{-1}$], calculated following, Pirt (1986) as *Y*_X,ph_ = μ_opt_ / (*q*_ph_ − *m*_ph_), varied 0.45, …, 0.73 ${\text{g}}_{\text{X}}{{\text{mol}}_{\text{ph}}}^{-1}$ during the experiments (based on Figure 3B), which matches the *Y*_X,ph_ range of 0.2, …, 2.1 ${\text{g}}_{\text{X}}{{\text{mol}}_{\text{ph}}}^{-1}$ reported in the literature (Zijffers et al., 2010; Dillschneider et al., 2013; Schediwy et al., 2019). The estimated parameter μ_max_ = 1.56 d^−1^ is comparable also to μ_max_ ranges between 0.86, …, 1.6 d^−1^ as found in the literature (Sandnes et al., 2005; Spolaore et al., 2006; Weise et al., 2019).

The similar parameters indicate that the model structure in particular with respect to the light-limited growth modeling shows a good transferability for tubular photobioreactors that differ in scale, glass tube diameter and the mode of bioprocess operation, although the experimental and modeling approaches differed in many respects. In contrast to Weise et al. (2019), who considered constant temperature conditions (*T* = 22 °C) under steady-state turbidostatic bioprocess operation, experiments presented here were conducted under variable temperature conditions and have been scaled up by ≈ 7x regarding the reactor liquid volume *V*_*L*_, by 10x regarding the number of modeled compartments and by 2x regarding the glass tube diameter.

In order to transfer the devised model to other reactors and different scales, it is necessary to consider homogeneous one-sided illuminated glass tube compartments within the model, although the glass tubes may be illuminated homogeneously from both sides in the actual arrangement. Therefore in this context also, the physical compartments are to be decomposed mathematically into several theoretical ones. If these assumptions do not apply to the specific photobioreactor structure, the model cannot be transferred without further modification. The presented model considers basic geometric properties (e.g., inner tube radius, liquid reactor volume, illuminated reactor surface, etc.) of tubular photobioreactors. Using biomass productivity or reactor size as scale-up objectives, it is possible to estimate the dimensions (e. g. illuminated reactor surface) of a photobioreactor scale-up.

The presented model is implemented using the ordinary differential equation (ODE) for batch bioprocesses. In order to adapt the model for continuous cultivation, the ODE for batch bioprocesses can be extended by a dilution term. The growth kinetics, which are described by algebraic equations are not affected by this extension.

**Figure 4:**
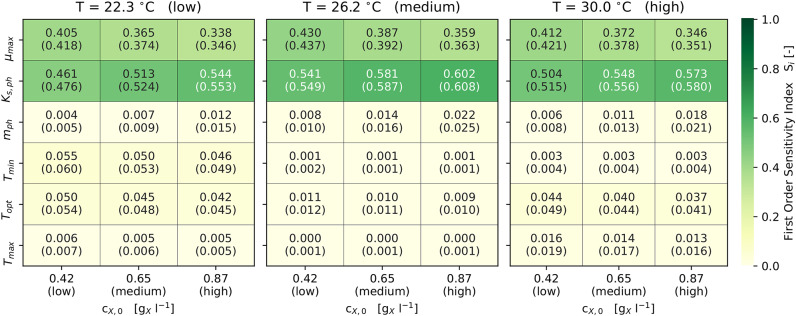
Results of the *Sobol* sensitivity analysis: First and (total) order sensitivity indices for Ω_f_ ± 3σ and 9 scenarios (3 each for *c*_X,0_ and *T*).

With respect to the model's sensitivity analysis, Figure 4 illustrates the first-order and total-order sensitivity indices of the model parameters across different scenarios. The comparatively small differences in the values of *S*_*i*_ and *S*_*Ti*_ indicate only limited interactions between the parameters, which was to be expected due to the model's structure (see Equations 1, 2, 7). The output of the model is highly sensitive (*S*_*i*_ and *S*_*Ti*_ ≥ 0.1) with respect to the parameters μ_max_ and *K*_S,ph_. Both parameters combined contribute predominantly to the variance in the model output, since they describe the utilization of light as the sole energy source for phototrophic growth. The set *c*_X,0_ effects the sensitivities of *K*_S,ph_ and *m*_ph_. Since higher *c*_X,0_ reduce the *q*_ph_, the photon half-saturation constant *K*_S,ph_ becomes more sensitive. In addition, *m*_ph_ becomes sensitive (*S*_*i*_ and *S*_*Ti*_ ≥ 0.01) only at higher *c*_X,0_ (Figure 4).

*T*_opt_ is sensitive (*S*_*i*_ and *S*_*Ti*_ ≥ 0.01) in all scenarios, while *T*_min_ and *T*_max_ are sensitive only at lower and higher temperatures. Also, *T*_min_ and *T*_max_ become non-sensitive at temperatures close to *T*_opt_. At temperatures away from *T*_opt_, the combined sensitivity of the three cardinal temperatures contributes up to 10 % (i.e., 0.1) to the model's output variance. The set *c*_X,0_ does not influence the sensitivity of the cardinal temperatures.

**Figure 5:**
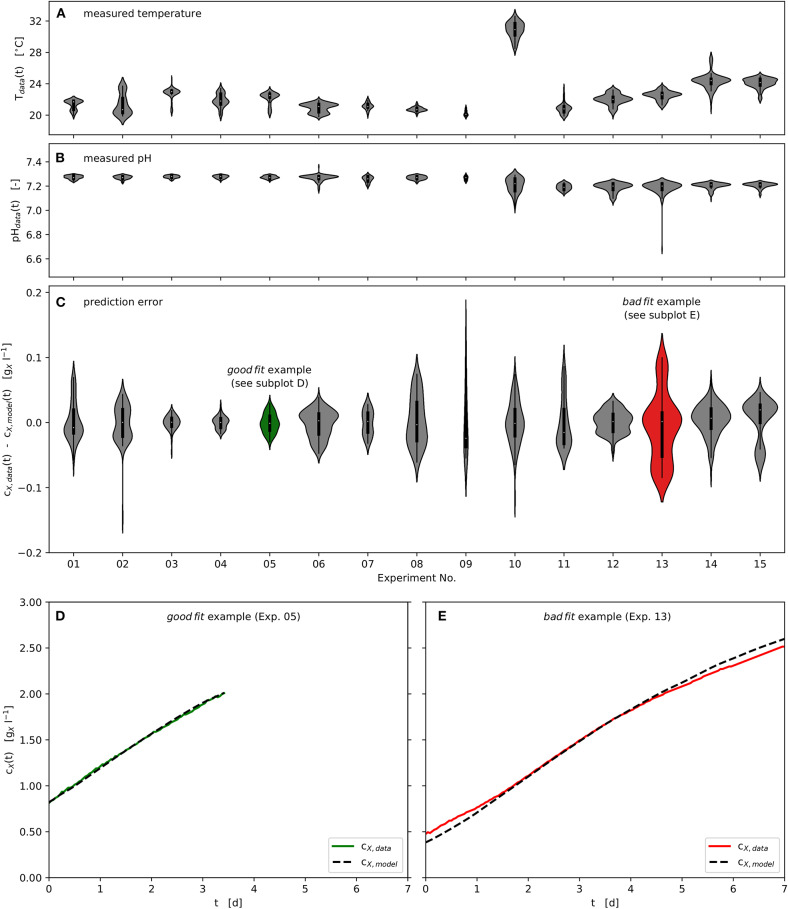
Experimental and modeling results: Time series of measured and modeled biomass concentration, measured temperature and pH; Violin plots: center point - median, box - interquartile range (IQR), whisker - 1.5 · IQR, kernel density estimation - number of observations; **(A)**: Temperature distributions of the experimental time series data; **(B)**: pH distributions of the experimental time series data; **(C)**: Model prediction error distributions of the experimental time series data with respect to biomass concentration; **(D)**: “good fit” example - representing lowest prediction error calculated, Experiment No. 05; **(E)**: “bad fit” example - representing highest prediction error calculated, Experiment No. 13.

All experiments were carried out in the range 20, …, 25°C, except Experiment No. 10 at 31°C. Although the temperature inside the photobioreactor was actively controlled, diurnal rhythms in the ambient temperature resulted in temperature variations within individual experiments. These are displayed in Figure 5A using kernel density approximations (violins). The width of the individual violin represents the number of available data at the respective temperature value, while small pin-like tails of the violins indicate short-term outliers of the measured variable. In analogy, Figure 5B shows the distribution of the measured pH values of the respective experiments. Variations in temperature and pH value did not occur rapidly, as can be seen in Figure 2. Despite the variations in the observed temperature and pH ranges, using the specific approach applied here, the available data could be successfully processed in order to estimate model parameters and optimize process parameters at pilot-scale. Figure 5C provides violin plots that represent the absolute modeling error with respect to the biomass concentration time series using the estimated parameter vector Ω_f_. It can be seen that the modeling error rarely exceeds ± 0.15 ${\text{g}}_{\text{X}}{\text{l}}^{-1}$.

Furthermore, one good as well as one badly predicted experiment are highlighted in *green* (Experiment No. 05) and *red* (Experiment No. 13), as shown in Figure 5C. These two experiments are presented as time series in Figures 5D,E. Although the biomass concentration has been well predicted over much of the time (Figures 5D,E), some regions are underestimated or overestimated at the beginning and the end of the respective experiments (Figure 5E as example).

The prediction accuracy MAPE (Equation 12) of the target value biomass productivity *Pr* (Equation 11) improved over the course of the estimation procedures. Table 2-lower part provides an overview of these experimental and modeling results as well as the parameters *a* and *b*. The *initial parameter estimation* of Ω_1_ and Ω_2_ resulted in MAPE(Ω_1_) = 7.5% and MAPE(Ω_2_) = 7.2%, whilst the prediction accuracy after the *final parameter estimation* was MAPE(Ω_f_) = 7.2%. Therefore, the accomplished overall model prediction accuracy is very satisfactory, although the *Absolute Percent Error* of individual experiments (APE_*j*_) covers a wider range 0.1, …, 26.1%. Typical MAPE values for industrial and business data and their interpretation are: < 10% highly accurate forecasting, 10, …, 20% good forecasting, 20, …, 50% reasonable forecasting, > 50% inaccurate forecasting (Lewis, 1982; Moreno et al., 2013).

Based on the results of the parameter estimation procedure (Table 2-upper part), the parameterized model has been used to design an optimized bioprocess regime regarding the inoculation biomass concentration *c*_X,0_, the cultivation cycle time *t*_cyc_ and the temperature *T* with respect to optimum biomass productivity *Pr*_opt_ for a series of equal batch cultivations (repeated batches). Since, the specific light availability rate *q*_ph_ decreases monotonously along with biomass accumulation under constantly illuminated discontinuous bioprocess operation, *q*_ph_ is only influenced by alterable *c*_X,0_ and *t*_cyc_.

Following the above objective, a numerical optimization of *Pr* was conducted using Equations (24) and (25) as described in section 2.6.6. The biomass productivity optimization showed two major aspects: First, the optimum growth temperature equals the model parameter *T*_opt_ = 27.93°C, which matches the intuitive expectation with regard to Equations (1) and (5). This applies independently of the lighting conditions according to the devised model. Second, the optimization results in a corner point optimum that points toward an insignificantly small cultivation cycle time *t*_cyc_ → 0.

The developed approach therefore predicts the theoretical optimum biomass productivity *Pr*_opt_ → 0.50 ${\text{g}}_{\text{X}}{\text{l}}^{-1}{\text{d}}^{-1}$ within the investigated bioreactor set-up for negligibly small cultivation cycle times with *c*_X,0,opt_ → 1.07 ${\text{g}}_{\text{X}}{\text{l}}^{-1}$, and hence the transition from discontinuous (e.g., batch, fed-batch or their repeated versions) to continuous (e.g., chemostat, turbidostat) bioprocess operation. The optimum inoculation biomass concentration *c*_X,0,opt_ calculated this way corresponds to the steady-state biomass concentration of a settled chemostat bioprocess or to the optimum biomass concentration set-point of a turbidostat bioprocess, respectively (Weise et al., 2019). (Statement is no longer applicable due to the modifiaction of Equation 2). The above findings are also supported by Chen et al. (2012) who estimated *c*_X,0,opt_ = 0.98 ${\text{g}}_{\text{X}}{\text{l}}^{-1}$ (*Pr*_opt_ = 0.75 ${\text{g}}_{\text{X}}{\text{l}}^{-1}{\text{d}}^{-1}$) for a small-scale laboratory set-up, as well as Hulatt et al. (2017) finding *Pr*_opt_ = 0.51 ${\text{g}}_{\text{X}}{\text{l}}^{-1}{\text{d}}^{-1}$ for a flat-panel photobioreactor, both under discontinuous operation. In addition, literature values for *Pr* at pilot-scale and industrial-scale show a range <0.1, …, 0.71 ${\text{g}}_{\text{X}}{\text{l}}^{-1}{\text{d}}^{-1}$ (de Vree et al., 2015; Pereira et al., 2018), confirming the feasibility of the *Pr*_opt_ values obtained here.

A simulation of the original experiments was carried out using the actually applied cultivation cycles times *t*_cyc_ of the experiments (see Table 2-lower part) as well as the optimized process parameters *c*_X,0,opt_ and *T*_opt_ (according to section 2.6.6). The model predicts a noticeable increase of *Pr* by 39% from 0.33 ${\text{g}}_{\text{X}}{\text{l}}^{-1}{\text{d}}^{-1}$ (see Table 2-lower part) to 0.46 ${\text{g}}_{\text{X}}{\text{l}}^{-1}{\text{d}}^{-1}$.

The convergence toward the predicted optimum biomass productivity is asymptotic. This results in only minor additional gains in biomass productivity when moving from short-cycled discontinuous to continuous bioprocesses and requires a simultaneous adaptation of the inoculation biomass concentration *c*_X,0_. Within this context, Table 3 shows examples of the predicted *Pr*_opt_ under discontinuous (repeated batch) bioprocess operation for fixed cultivation cycle times *t*_cyc_ (1 d, 3.5 d, and 7 d) starting at the required inoculation biomass concentration *c*_X,0_ and under optimum temperature *T*_opt_. As a consequence, solely reducing *t*_cyc_ from 7 d to 3.5 d would outweigh the increase in operational efforts by a higher *Pr* (0.37 → 0.46 ${\text{g}}_{\text{X}}{\text{l}}^{-1}{\text{d}}^{-1}$; with *c*_X,0,opt_ 0.17 → 0.43 ${\text{g}}_{\text{X}}{\text{l}}^{-1}$) under discontinuous bioprocess operation.

**Table 3:** Numerical optimization results: Model predicted *Pr*_opt_ for different bioprocess operations and pre-set *t*_cyc_ at *T*_opt_ with the required *c*_X,0,opt_ (see Figure 6A).

**Bioprocess operation**	***t*_cyc_**	***c*_X,0,opt_**	***Pr*_opt_**
	**[d]**	**[g_**X**_ l^**−1**^]**	**[g_**X**_ l^**−1**^ d^**−1**^]**
Discontinuous (repeated)	7.0	0.17	0.37
Discontinuous (repeated)	3.5	0.43	0.46
Discontinuous (repeated)	1.0	0.84	0.50
Continuous	→ 0	1.07	0.50

**Figure 6:**
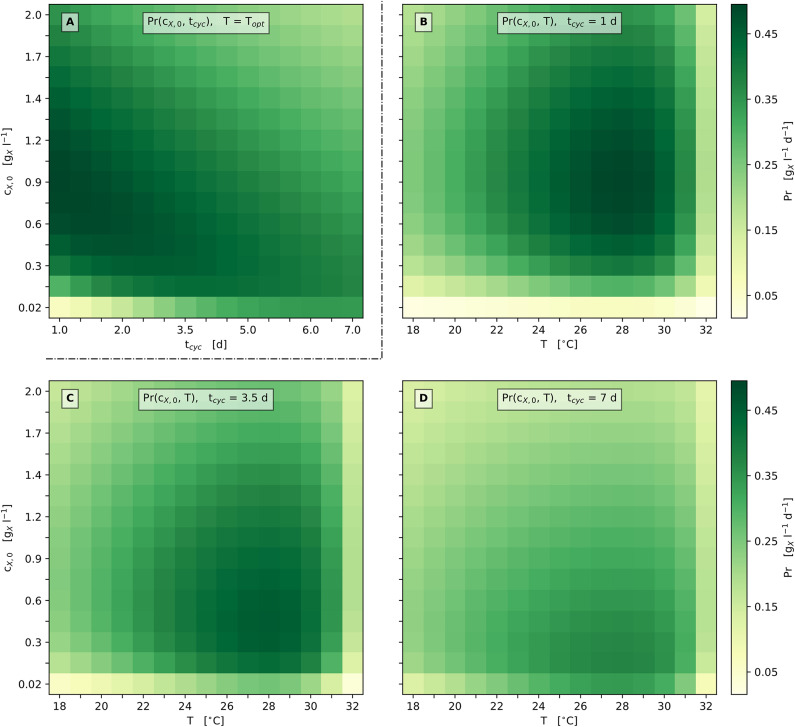
Heat map representation of model predicted biomass productivity; **(A)**: Pr depending on *t*_cyc_ and *c*_X,0_ at *T*_opt_; **(B–D)**: Pr depending on *T* and *c*_X,0_ at set *t*_cyc_ [1 d **(B)**, 3.5 d **(C)**, 7 d **(D)**].

Figure 6A illustrates these relations in more detail by presenting the model's prediction with respect to biomass productivity *Pr* depending on the cultivation cycle time *t*_cyc_ and the inoculation biomass concentration *c*_X,0_ for the optimum cultivation temperature *T*_opt_. According to the model's prediction, only moderately high biomass productivities are gained for cultivation cycle times greater ≈ 5 days, as well as for inoculation biomass concentrations above ≈ 1 ${\text{g}}_{\text{X}}{\text{l}}^{-1}$ (Figure 6A). Notably, the optimum productivity is reached at the shortest cultivation cycle time, which illustrates the said corner point optimum regarding the cultivation cycle time *t*_cyc_.

In accordance with these findings, Figures 6B–D additionally provides graphical representations of the biomass productivity loss when varying the process parameters *c*_X,0_, *t*_cyc_ and *T*. Figures 6B–D illustrate scenarios for three different cultivation cycle times: 1 d, 3.5 d (1/2 week), and 7 d (1 week) with respect to discontinuous bioprocess operation (repeated batch). Considerations regarding lag phases are neglected here, as can be seen in Figures 2, 5D,E. All three scenarios show a single optimum of biomass productivity at the respective optimum temperature *T*_opt_ and the optimum inoculation biomass concentration *c*_X,0,opt_.

The predicted *Pr*_opt_ increases by about 35% from 0.37 ${\text{g}}_{\text{X}}{\text{l}}^{-1}{\text{d}}^{-1}$ at *t*_cyc_ = 7 d (*c*_X,0,opt_ = 0.17 ${\text{g}}_{\text{X}}{\text{l}}^{-1}$) to 0.50 ${\text{g}}_{\text{X}}{\text{l}}^{-1}{\text{d}}^{-1}$ at *t*_cyc_ = 1 d (*c*_X,0,opt_ = 0.84 ${\text{g}}_{\text{X}}{\text{l}}^{-1}$). In general, the optimum biomass productivity *Pr*_opt_ increases when the cultivation cycle time is shortened. In addition, when this time is shortened, a higher *c*_X,0,opt_ is required to obtain the optimum productivity.

## 4. Conclusion

The work presented here provides a transferable methodology to model microalgal growth covering light availability and temperature based on experimental data from cultivation runs in a small pilot-scale tubular photobioreactor under discontinuous operation in order to subject it to biomass productivity analysis and optimization.

The established model with its estimated parameters accurately predicts light and temperature dependent growth of *Nannochloropsis granulata*. The parameter ranges are supported by the literature. In general, the model parameter *T*_opt_ is much closer to *T*_max_ than to *T*_min_, thus the CTMI displays a strong asymmetry. Temperatures above *T*_opt_ therefore lead to a steep decline in the growth rate and also the biomass productivity. Since an accurate temperature control is hardly to provide under large-scale or outdoor conditions, these processes should be operated below the targeted *T*_opt_.

Model-based numerical biomass productivity optimization for repeated discontinuous operation points toward best performance under continuous operation. The optimization for repeated discontinuous operation yields reduction of cultivation cycle time and increase of inoculation biomass at optimum temperature. The calculated optimum inoculation biomass concentrations *c*_X,0,opt_ and the corresponding optimum biomass productivities *Pr*_opt_ are confirmed by different publications. Furthermore, biomass productivities for laboratory-scale, pilot-scale and industrial-scale reported in the literature support the feasibility of the *Pr*_opt_ values obtained here. Applying these optimized process parameters would deliver a noticeable increase in biomass productivity.

The successful application of this approach here to small pilot-scale under discontinuous operation, following a previous investigation into lab-scale under continuous operation, indicates its potential transferability also to larger scale tubular photobioreactors covering both light and temperature dependent microalgal growth and biomass productivity.

## Data Availability Statement

All datasets generated for this study are included in the article/Supplementary Material.

## Author Contributions

TW contributed to the conception and design of the study, the collection and assembly of data, the analysis and interpretation of the data, the drafting, critical revision and final approval of the article. CG contributed to the collection and assembly of data, the drafting, critical revision and final approval of the article and to obtaining funding. MP contributed to the conception and design of the study, the interpretation of the data, the drafting, critical revision and final approval of the article and to obtaining funding.

## Conflict of Interest

TW is collaborative doctoral candidate at the University of Applied Sciences Jena, Jena, Germany and the Friedrich Schiller University Jena, Jena, Germany. TW was employed by the University of Applied Sciences Jena, Jena, Germany and is now employed by the company BioControl Jena GmbH, Jena, Germany. CG is employed by the company Salata AG, Ritschenhausen, Germany. MP is professor at the University of Applied Sciences Jena, Jena, Germany. MP owns stock in the company BioControl Jena GmbH, Jena, Germany.

## Figures and Tables

**Table:** 

**Symbol**	**Description**	**Unit**
***Latin***		
*A*	Illuminated photobioreactor projection surface	m^2^
*c*	Number of photobioreactor compartments	−
*c*_X_	Biomass concentration (within the photobioreactor)	${\text{g}}_{\text{X}}{\text{l}}^{-1}$, ${\text{g}}_{\text{X}}{\text{m}}^{-3}$
*I*_0_	Light intensity at the photobioreactor surface	${\text{mol}}_{\text{ph}}{\text{m}}^{-2}{\text{d}}^{-1}$
*J*	Cost function	−
*K*_S,ph_	Specific photon half-saturation constant	${\text{mol}}_{\text{ph}}{{\text{g}}_{\text{X}}}^{-1}{\text{d}}^{-1}$
*l*	Light path length	m
*l*_∅_	Average light path length	m
*m*_ph_	Specific photon maintenance coefficient	${\text{mol}}_{\text{ph}}{{\text{g}}_{\text{X}}}^{-1}{\text{d}}^{-1}$
*M*	Model output	
(M)APE	(Mean) absolute percent error	%
*OD*_NIR_	Optical density at near infrared range	AU
*Pr*	Biomass productivity	${\text{g}}_{\text{X}}{\text{l}}^{-1}{\text{d}}^{-1}$
*Pr*_opt_	Optimum biomass productivity	${\text{g}}_{\text{X}}{\text{l}}^{-1}{\text{d}}^{-1}$
*q*_ph_	Specific photon availability rate	${\text{mol}}_{\text{ph}}{{\text{g}}_{\text{X}}}^{-1}{\text{d}}^{-1}$
*r*	Inner radius of tube/compartment	m
*S*	*Sobol* sensitivity index	–
*S*_*T*_	*Sobol* total sensitivity index	–
*t*	Time	d
*T*	Temperature (within the photobioreactor)	°C
*V*_*L*_	Liquid volume of the photobioreactor	m^3^
*Var*	Variance	
*Y*_X,ph_	True photon growth yield	${\text{g}}_{\text{X}}{{\text{mol}}_{\text{ph}}}^{-1}$
***Greek***		
μ	Specific growth rate	d^−1^
μ_max_	Maximum specific growth rate	d^−1^
ϕ(*T*)	Cardinal temperature model with inflexion (CTMI)	−
$\hat{\phi }\left(T\right)$	Cardinal temperature model with inflexion (CTMI) for *T*_min_ < *T* < *T*_max_	−
σ	Standard deviation	
θ	Vector of all possible parameter combinations	
Ω	Vector of estimated optimum parameter values	
***Indices***		
0	Value at begin of cultivation	
cyc	Cultivation cyle	
f	Final value	
min/max	Minimum/maximum value	
meas/data	Measured value	
opt	Optimum value	
ph	Photon	
pred/model	Predicted/modeled value	
X	Biomass	
∅	Average value	
